# Conditioned Fear Associated Phenotypes as Robust, Translational Indices of Trauma-, Stressor-, and Anxiety-Related Behaviors

**DOI:** 10.3389/fpsyt.2014.00088

**Published:** 2014-07-21

**Authors:** Maria Anne Briscione, Tanja Jovanovic, Seth Davin Norrholm

**Affiliations:** ^1^Trauma Recovery Program, Mental Health Service Line, Atlanta Veterans Affairs Medical Center, Decatur, GA, USA; ^2^Department of Psychiatry and Behavioral Sciences, Emory University School of Medicine, Atlanta, GA, USA

**Keywords:** fear learning, startle reaction, anxiety disorders, traumatology, translational medical research

## Abstract

Post-traumatic stress disorder (PTSD) is a heterogeneous disorder that affects individuals exposed to trauma (e.g., combat, interpersonal violence, and natural disasters). It is characterized by hyperarousal, intrusive reminders of the trauma, avoidance of trauma-related cues, and negative cognition and mood. This heterogeneity indicates the presence of multiple neurobiological mechanisms underlying the development and maintenance of PTSD. Fear conditioning is a robust, translational experimental paradigm that can be employed to elucidate these mechanisms by allowing for the study of fear-related dimensions of PTSD (e.g., fear extinction, fear inhibition, and generalization of fear) across multiple units of analysis. Fear conditioning experiments have identified varying trajectories of the dimensions described, highlighting exciting new avenues of targeted, focused study. Additionally, fear conditioning studies provide a translational platform to develop novel interventions. The current review highlights the versatility of fear conditioning paradigms, the implications for pharmacological and non-pharmacological treatments, the robustness of these paradigms to span an array of neuroscientific measures (e.g., genetic studies), and finally the need to understand the boundary conditions under which these paradigms are effective. Further understanding these paradigms will ultimately allow for optimization of fear conditioning paradigms, a necessary step towards the advancement of PTSD treatment methods.

## Introduction

Post-traumatic stress disorder (PTSD) affects 8% of the general population and occurs at a much higher percentage in populations at risk of experiencing trauma; this includes military personnel and individuals living in low-socioeconomic urban environments (1, 2). The incidence of combat-related PTSD is expected to rise given the number of veterans returning from theaters of conflict in Iraq and Afghanistan. According to early reports, approximately 20% of Operation Iraqi Freedom and Operation Enduring Freedom (OEF and OIF, respectively) veterans presented with PTSD symptoms upon their return from combat (3). While these recent conflicts have generated new cases of PTSD, there remain a significant number of Vietnam veterans who have been experiencing persistent PTSD symptoms for as long as 40 years (4). Based on earlier work from our group, PTSD is equally as pervasive in low-socioeconomic urban environments including Atlanta, GA, USA (5, 6).

Post-traumatic stress disorder is the fifth most common psychiatric diagnosis and is not limited to the aforementioned groups (2, 7). Unfortunately, many of the traumatic events that precede the development of PTSD are not preventable, and we can expect new cases to develop as a result of widespread trauma across military and civilian populations. For example, returning veterans from the most recent combat theaters have been exposed to the unpredictable nature of urban warfare, which includes suicide bombings and improvised explosive devices (IEDs). In civilian populations from low-socioeconomic urban environments, there is risk of interpersonal violence and sexual assault. Further, victims of natural disasters, who can number in the thousands, can develop PTSD symptoms in the wake of unforeseen devastation to person and property (8). It is becoming increasingly clear that one's individual risk for developing PTSD following exposure to a traumatic event is influenced by both intrinsic (e.g., genomic) and extrinsic (e.g., social support network) factors.

Post-traumatic stress disorder is a heterogeneous disorder in which symptoms span four primary symptom clusters according to the recent *Diagnostic and Statistical Manual of Mental Disorders*, 5^th^ edition [DSM-5; (9)]. The DSM-5 PTSD symptoms clusters include: (1) re-experiencing, such as flashbacks and recurrent nightmares, (2) avoidance, which includes circumventing thoughts and feelings associated with the traumatic event, (3) negative cognitions and mood, which encompasses detachment from others and a loss of interest in activities, and (4) hyperarousal, which can manifest itself as difficulty sleeping and feeling overly alert.

This heterogeneity implies the involvement of multiple neurobiological mechanisms, which underlie the development and maintenance of PTSD [for a comprehensive review, see (10)]. The classification of PTSD symptoms according to clusters provides some utility in the clinic; however, there is a movement in the field to adopt a new strategy focused on the study of neurobiological mechanisms that “cut across” mental disorders and underlie multiple psychiatric disease states [Research Domain Criteria; RDoC; (11)]. A central tenet of the RDoC framework is “classifying psychopathology based on dimensions of observable behavioral and neurobiological measures.” RDoC focuses on several different systems, including the Negative Valence System; the construct matrix for this system includes acute threat or fear, which can be measured across several units of analysis. The focus of this review is on fear-conditioning studies that are defined under this construct.

Fear conditioning paradigms provide a compelling translational platform for investigating the neural underpinnings of trauma- and stressor-related disorders, such as PTSD and anxiety disorders such as panic and specific phobia. Interestingly, the DSM-5 no longer includes Criterion A2, or the presence of fear, helplessness, or horror in response to a traumatic event, in the diagnostic criteria that must be endorsed for a clinical diagnosis of PTSD; however, dysregulation of fear conditioning-related phenotypes remains a central feature of this disorder (12). As described in a recent review by Weston (13), neural circuitry that includes the amygdaloid complex can be associated with at least 14 symptoms of PTSD and, as such, there remains compelling interest in developing and utilizing translational paradigms that index these circuits, including fear inhibition (14), fear extinction (15), and stimulus generalization (16). In translational experimental paradigms, the general term extinction can refer to the learning process that occurs during the non-reinforced presentation of a previously reinforced CS (termed extinction training) as well as the retention of extinction learning after a period of time has elapsed since extinction training [termed extinction recall; (17)].

KEY CONCEPT 1. Fear conditioning A paradigm where a neutral stimuli is paired with unpleasant/aversive event to conceptualize and objectively study a traumatic experience according to the principles of Pavlovian conditioning such that the unconditioned fear responses are similar to those experienced at the time of trauma that become elicited by stimuli similar to those present at the time of the trauma.

KEY CONCEPT 2. Fear inhibition Fear inhibition refers to the ability to inhibit a fear response in the presence of a safety signal and can be observed experimentally when a previously reinforced CS⩲ is presented in compound with a neutral, safe stimulus.

KEY CONCEPT 3. Fear extinction Fear extinction is a form of new learning that occurs when the previously reinforced CS⩲ is repeatedly presented in the absence of the aversive US. It is a translational tool, such that it is experimentally homologous to exposure therapy. Dysregulated fear extinction in patients with PTSD appears to manifest itself in at least three ways that may not be mutually exclusive.

## Fear Conditioning

Self-report measures of PTSD can often be subjective and unreliable. The high comorbidity of PTSD and depression highlights this problem; specifically, measures of PTSD and depression [as indexed by the Clinician Administered PTSD Scale (CAPS) and the Beck Depression Index (BDI), respectively) may reflect overlapping symptoms of negative affect (18, 19). Similarly, the symptom dimensions used for a diagnosis may reflect complex sequelae resulting from an overlap in symptom presentation of multiple disorders (20). Fear conditioning allows the use of quantitative objective measures to identify and differentiate components of PTSD, most notably those related to the dysregulation of fear processing.

Fear conditioning utilizes Pavlovian conditioning and involves the association of previously neutral stimuli with unpleasant or aversive events. Experimentally, a neutral stimulus (i.e., shape or sound) is spatially or temporally paired with an aversive unconditioned stimulus (US), typically a blast of air or a mild electric shock. Previously, the neutral stimulus comes to evoke the same response as the US, even in the absence of the US, and is termed the reinforced conditioned stimulus [CS+, Ref. (21)]. When conceptualizing a traumatic experience according to the principles of fear conditioning, unconditioned fear responses (UCR) similar to those experienced at the time of trauma can subsequently be elicited by stimuli (termed conditioned stimuli or CSs) similar to those present at the time of the trauma (e.g., sights, sounds, smells, context).

Conditioned fear responses can be quantified experimentally in a variety of ways. Fear potentiation of the acoustic startle response is a commonly employed translational methodology for indexing learned fear. The acoustic startle response (“startle”) is characterized by an integrative reflex contraction of the skeletal musculature in response to a strong stimulus (e.g., loud noise) and is an ideal model for studying fear conditioning since the amygdala is directly inter-connected with the startle circuit (22–24). Fear-potentiated startle is defined as an increase in the magnitude of the startle response when it is elicited in the presence of a CS+ that has been repeatedly paired with an aversive US; this methodology inherently includes a within-subject non-zero baseline measure of an individual's acoustic startle response (25, 26). Fear-potentiated startle is observed across species and is ideal for studying translational models of fear-related phenotypes (27).

KEY CONCEPT 4. Fear-potentiated startle Conditioned fear responses can be quantified experimentally in a variety of ways and fear potentiation of the acoustic startle response is a commonly employed translational methodology for quantitatively indexing learned fear across mammalian species. It refers to the relative increase from baseline startle in the presence of a fearful stimulus.

In typical human fear-conditioning approaches, an acquisition phase is presented in which a previously neutral CS is paired with an aversive US such that the CS comes to predict the US and, as such, a fear response of interest is elicited (potentiation of startle as compared to baseline or increase in skin conductance in the presence of the CS+ as opposed to a non-reinforced CS−). In addition, many paradigms will include a real-time measure of US-expectancy such that participants report their prediction of the presence or absence of a CS on a trial-by-trial basis. This allows investigators to determine whether participants can accurately discriminate between danger cues (CS+) and safety cues (CS). These measures further allow investigators to discern a psychophysiological, but not a cognitive, response consistent with a dysregulated system. For example, PTSD patients have shown increased fear responses to cues that they subjectively report as safe (28).

**Figure 1:**
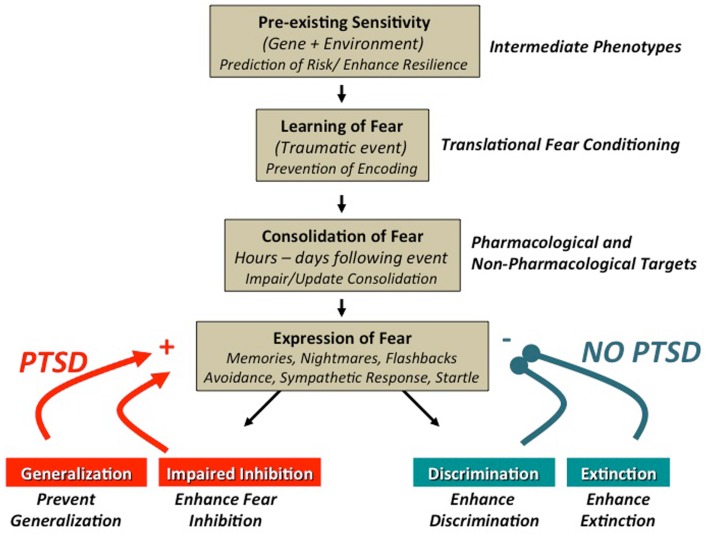
**Promising targets for the translational study of trauma-, stressor-, and anxiety-related fear behaviors: trauma-, stressor-, and anxiety-related disorders have been shown to have a significant degree of heritability and more recently, it has become increasingly clear that genetic contributions include complex gene × environment interactions**. Understanding these complex relationships may allow for early interventions to enhance resiliency in certain individuals with a high risk of trauma. Fear-conditioning paradigms afford the study of intermediate phenotypes, which may enhance our ability to elucidate these complex interactions [see Ref. (36)]. The dysregulated fear learning commonly observed in anxious and traumatized populations can be modeled by fear-conditioning paradigms, which can provide a translational framework. Translational studies have shown that fearful memories are initially labile and are consolidated to a more permanent state, hours to days, after the initial event. Modeling the process of consolidation and reconsolidation with fear-conditioning techniques provides an avenue to study potential pharmacological and non-pharmacological interventions [e.g., Ref. (33, 37)]. Clinically, individuals with PTSD have been show to have alterations in stimulus generalization (16), fear inhibition (29), discrimination, and fear extinction (38). In addition to complementary psychophysiological methods (e.g., skin conductance, reaction time), fear-potentiated startle methods have proven to be quite useful for the study and/or manipulation of these targets to better understand and treat stressor-, trauma-, and anxiety-related disorders. Figure adapted from Jovanovic and Ressler (39).

Following an Acquisition phase, translational studies of conditioned fear will often employ one of three procedures for measuring the expression and/or inhibition of the newly acquired fear: (1) fear extinction, (2) fear inhibition, or (3) generalization of fear. Alterations in fear extinction, fear inhibition, and stimulus generalization have all been reported, using fear-potentiated startle methods, in populations with trauma- and stressor-related as well as anxiety disorders [see Figure 1; (16, 29–31)]. Fear extinction is a form of new learning that occurs when the previously reinforced CS+ is repeatedly presented in the absence of the aversive US [e.g., Ref. (17)]. Despite some recent reports to the contrary [e.g., Ref. (32, 33)], the predominant understanding is that the original fear memory (CS–US association) is not erased, but competes with new extinction learning, and can be accessed through the processes of spontaneous recovery (following the passage of time), renewal (shift in context), or reinstatement (the unsignaled presentation of the aversive US). Fear inhibition has traditionally been observed when a previously reinforced CS+ (termed cue A) is presented in compound with a neutral stimulus [termed the AB compound; (34)]. More recent studies of fear inhibition have used compound stimuli in which one compound is reinforced (termed AX+), another is non-reinforced (termed BX−) during acquisition. Fear inhibition is then measured as the transfer of the inhibitory (safe) properties of stimulus B onto stimulus A [via presentation of an AB compound; (29, 35)]. Generalization of fear (or stimulus generalization) has been more widely studied recently and is a measure of the degree to which a conditioned fear response is expressed in the presence of generalization stimuli (GSs) that vary incrementally from an initially presented CS+ [e.g., concentric rings of increasing or decreasing diameter from the original CS+; (27); or morphed faces of which one is the CS+; (31)].

KEY CONCEPT 5. Generalization of fear Generalization of fear or stimulus generalization refers to the ability to discern fearful and safety cues. Generalization is a measure of the degree to which a conditioned fear response is expressed in the presence of generalization stimuli that vary incrementally from an initially presented CS⩲.

Fear extinction, as a translational tool, is an important area of study due to its relevance to the limbic neural circuitry believed to underlie fear psychopathology and also due to its role as an experimental homolog to exposure therapy, which is currently recognized as the most effective treatment for fear and anxiety. Dysregulated fear extinction in patients with PTSD symptoms appears to manifest itself in at least three ways that may not be mutually exclusive: over-expression of acquired fear, impaired within-session extinction learning (decrease in fear observed during the experimental session), and impaired between-session extinction retention (decrease in fear from one session to the next, which reflects memory consolidation processes). With regard to over-expression of acquired fear, previous work from our lab (15) suggests that the early phase of within-session extinction learning involves persistent excitation, as it is predicted by the level of fear expression to the CS+ (i.e., the danger signal) at the end of acquisition. For example, our group showed heightened levels of fear during early extinction in a previously traumatized population with PTSD consisting of primarily African-American women living in a low-socioeconomic urban environment; we termed this pattern of extinction learning as being indicative of “fear load,” or the over-expression of fear during early extinction. High levels of conditioned fear remaining during late extinction are related to impaired inhibition, as it is best predicted by responses to the CS− (i.e., safety signal) at the end of acquisition (15). This is based on the notion that extinction learning is a form of fear inhibition. As an example, we previously showed that a predominately male population of combat veterans with PTSD did not show “fear load,” but rather impaired within-session extinction learning characterized by a “persistence of fear” (40). Lastly, work by Milad and others (41, 42) demonstrated that individuals with PTSD showed a reduced ability to recall extinction learning when tested 24 h after within-session extinction learning had occurred. The presence of divergent extinction trajectories as described above represents an exciting new avenue of study and has recently been introduced in the rodent literature (43), which may be analogous to heterogeneous responses to trauma in PTSD (44).

## Units of Analysis

While PTSD symptom clusters provide a useful set of diagnostic criteria and could be used to discern the most effective treatment [for a comprehensive review, see Ref. (10)], they represent broad categories of behavior and do not easily reflect common underlying mechanisms. Using the RDoC approach, fear-conditioning studies can identify intermediate phenotypes, which represent specific components of a disorder and allow for a more direct examination of brain–behavior relationships. We define intermediate phenotypes as observable units that are (1) related to the underlying neurobiology of a disorder, (2) related to clinical symptoms of the disorder, and (3) are ideally possible to model in animal studies affording a translational approach. Unlike an endophenotype, an intermediate phenotype does not necessarily require heritability. An intermediate phenotype can be assessed with different units of analysis, including molecular, neural circuitry, physiology, behavior, and self-reports such as those listed in the RDoC matrix (http://www.nimh.nih.gov/research-priorities/rdoc/nimh-research-domain-criteria-rdoc.shtml). For the purpose of this review, we focused on physiological units of analysis, including fear-potentiated startle and skin conductance response as fear conditioning-related intermediate phenotypes.

KEY CONCEPT 6. Fear conditioning-related intermediate phenotypes Observable units that are (1) related to the underlying neurobiology of a disorder, (2) related to clinical symptoms of the disorder, and (3) are ideally possible to model in animal studies affording a translational approach.

Numerous translational studies have employed fear-conditioning paradigms to better identify more basic dimensions and phenotypes associated within these diagnostic criteria. For example, previous work has shown that subjects with high hyperarousal symptoms show the greatest difficulty inhibiting a fear response to safety cues (45). In addition, during extinction learning, increased fear-potentiated startle was associated with re-experiencing symptoms of PTSD (15). The same study showed a more robust fear response to both the CS+ (danger cue) and the CS− (safety cue) during acquisition that was associated with higher re-experiencing and hyperarousal symptoms.

Research has indicated that fear conditioning and cognitive biases share a common underlying neural mechanism, amygdala-prefrontal circuitry (46). One aspect of cognitive bias, attention bias, or the facilitated orientation toward, or avoidance of, specific cues, has been shown to be a useful index of anxiety-related disorders (46). This proposed connection between attention bias and fear conditioning has been explicitly examined. Fani et al. (47) showed that attention bias toward threat is associated with over-expression of fear during early extinction, referred to as “fear load.” This study provides further evidence of the utility of intermediate phenotypes to enhance our understanding of underlying neural circuitry and corresponding behavioral responses.

Further, PTSD has long been associated with intrinsic [e.g., age and gender (48, 49)], environmental (e.g., exposure to trauma, rearing environment, degree of social support), and genetic factors (50–52). The dysregulated fear learning observed in subjects with PTSD likely reflects the complexity of these interactions. Specifically, several candidate gene studies have identified genetic differences and specific gene pathways involved in PTSD [for a review, see (53)]. When conducting both candidate gene studies and genome-wide association studies (GWAS), it is common for the resulting genetic findings to associate with many disorders or only to distinct phenotypes of broader disorders, a central concern outlined in RDoC. It is useful to instead study intermediate phenotypes or specific components of disorders. For example, we recently showed that increased “fear load,” as measured by fear-potentiated startle was related to the catechol-*O*-methyl-transferase (COMT) Val66Met polymorphism and a diagnosis of PTSD (36). More recently, GWAS data, which is obtained when expression levels of the whole genome are analyzed in the context of PTSD, have been used to identify novel gene pathways involved in the mechanisms underlying complex disorders such as PTSD [see Ref. (54) for a review]. The use of these intermediate phenotypes not only enhances the ability to identify complex gene interactions but also allows for these gene pathways to be more readily studied. Fear conditioning provides a potential framework for studying these phenotypes [see Ref. (55, 56)].

## Versatility

Fear-potentiated startle can be used in several mammalian species (57); however, in animal models the CSs are typically auditory, whereas visual CSs are used in human studies. In order to bridge this gap, Norrholm and colleagues examined the use of auditory and visual CSs in fear conditioning of healthy participants (58). Briefly, healthy participants acquired fear to auditory stimuli comparable to the participants who were fear conditioned with visual stimuli. Both groups also discriminated between the CS+ and CS−; however, the auditory group exhibited discrimination on blocks 1, 2, and 3 of fear acquisition, while the visual CS+ group exhibited discrimination on blocks 2 and 3 of fear acquisition. Ten minutes after this initial assessment, these groups were shown to also extinguish fear; however, the auditory group displayed a steeper slope of extinction, due in part to an initial increase in fear during extinction, than the visual group. Twenty-four hours later an extinction test was performed. This test showed that spontaneous recovery occurred in both groups and US-expectancy ratings increased in both groups regardless of modality as well (58). The advantage of this versatility is that it can be used in patients with visual impairment with equivalent results and also lends itself to the use of “cross-over” longitudinal studies in which practice effects can be minimized.

## Treatment

Not surprisingly, treatment approaches aimed at reducing the fear-related symptoms of PTSD (e.g., re-experiencing and intrusive memories) have focused on the disruption of fear memory consolidation/reconsolidation, facilitation of extinction learning, and the prevention of the return of fear. Several recent translational studies have explored both pharmacological as well as non-pharmacological means of enhancing extinction of fear and preventing its re-emergence. Memory consolidation refers to the process of transforming a memory (e.g., fear memory) from a labile state immediately after acquisition to a more permanent state that occurs after some time [believed to be 6 h or more post-acquisition; e.g., Ref. (59)]. Clinically speaking, memory consolidation first occurs at the time at which the traumatic event occurs. Many cellular processes have been described as underlying the neural mechanisms of consolidation, including the activation of β-adrenergic receptors in amygdala; however, administration of the β-adrenergic receptor antagonist, propranolol, while initially promising, has not shown any significant effects regarding memory consolidation in large scale studies (60, 61). Morphine has also been studied as a possible intervention of fear memory consolidation; however, it is not clear whether morphine's analgesic properties act to reduce the potency of the CS+ or are acting through a separate mechanism (61, 62).

KEY CONCEPT 7. Memory consolidation Memory consolidation is the process of transforming a memory (e.g., fear memory) from a labile state immediately after acquisition to a more permanent state that occurs after sometime. This process has been the target for non-pharmacological attempts to facilitate fear extinction and prevent the return of conditioned fear in humans.

With regard to the facilitation of extinction learning, a potential avenue was revealed by Davis and colleagues who reported that learning to extinguish conditioned fear was dependent on *N*-methyl-d-aspartate (NMDA) glutamate receptors in limbic regions including the amygdala (63). A decade later, Walker et al. (64) found that d-cycloserine (DCS), an NMDA receptor partial agonist, facilitated extinction learning in rats; a finding that was later replicated by several other groups employing multiple types of fear learning paradigms in rodents [e.g., Ref. (65)] but not replicated using skin conductance and expectancy ratings in humans (66). Shortly thereafter, several groups reported that DCS administration before or after exposure therapy, a form of treatment based on the principles of fear extinction learning, was effective in alleviating the symptoms of acrophobia (67), panic disorder (68), social anxiety disorder (69, 70), and obsessive–compulsive disorder (71–73).

At present, the effectiveness of DCS in facilitating exposure therapy (i.e., extinction learning) for PTSD is unclear due to mixed reports in the extant literature. DCS has been shown to be effective under specific clinical conditions. For example, de Kleine and colleagues reported increased improvement in the symptom ratings of patients who were administered DCS and initially reported more severe symptom severity (74). Initial reports by Litz et al. (75) suggested that DCS + exposure therapy was not as effective as placebo + exposure in a population of combat veterans with PTSD (75) However, more recent reports from the latter group suggest that the effectiveness of DCS as an adjunct therapy may be related to the degree of fear reduction observed during individual exposure therapy sessions (76, 77) Additionally, a recent study used DCS in addition to virtual reality exposure therapy in patients suffering PTSD as a result of the 9/11 attacks and found significant clinical advantages of DCS compared to placebo (78). Specifically, when DCS was administered prior to virtual reality exposure therapy subjects showed earlier, enhanced symptom reduction and greater PTSD remission rates (78). Finally, trauma imagery-potentiated startle responses in recently returned combat veterans from Iraq and Afghanistan were significantly reduced in individuals who previously received DCS before each of five sessions of virtual reality exposure therapy for PTSD (38).

In addition to agents that act on glutamatergic systems, other pharmacological approaches have been examined as possible therapies for fear- and anxiety-related disorders because of their potential to facilitate extinction learning and/or prevent the return of fear. Pre-clinical rodent studies have shown that monoaminergic specific antidepressants such as venlafaxine (serotonin–norepinephrine reuptake inhibitor; SNRI) and fluoxetine (serotonin specific reuptake inhibitor; SSRI) can facilitate between-session extinction (79, 80) and prevent reinstatement of conditioned fear (80, 81). Although the use of antidepressants has not been widely studied in extinction of fear-potentiated startle studies, there is some evidence from skin conductance-based investigations suggesting a facilitatory role of these drugs in fear extinction learning (82).

The endocannabinoid system has also been implicated in conditioned fear extinction and represents an additional area of exploration for facilitating extinction and prevention of fear return. Chhatwal et al. (83) showed that enhanced cannabinoid receptor CB1 activation (via administration of AM404, an agent that prevents endocannabinoid degradation and reuptake) facilitated within-session and between-session extinction of fear-potentiated startle as well as reinstatement in mice (83). This work was furthered by Gunduz-Cinar et al. (84, 85) who showed that inhibition of fatty acid amide hydrolase (FAAH), the enzyme that catabolizes the endogenous cannabinoid anandamide, enhances extinction learning in rodents and could represent a risk variant for stressor-related psychiatric disorders (84, 85).

New avenues for exploration continue to emerge and represent expanding opportunities to apply the fear conditioning and extinction procedures described herein. For example, Acheson and colleagues showed that the extinction of fear-potentiated startle in healthy humans could be enhanced with the intranasal administration of oxytocin (86). In addition, recent evidence from mouse studies implicates the renin–angiotensin system in the regulation of fear and anxiety responses as angiotensin receptor AT1 antagonism with the anti-hypertension drug losartan has been shown to enhance fear extinction learning [extinction recall; (87)].

Non-pharmacological attempts to facilitate fear extinction and prevent the return of conditioned fear in humans have focused on the disruption of the original fear memory trace (CS–US association) by interfering with consolidation or reconsolidation following retrieval of a previously stored fear memory. One approach for disrupting consolidation of fear memories (CS–US association) that has been explored is immediate versus delayed extinction training. Myers et al. (32) showed that extinction training initiated immediately after (10 min) fear acquisition prevented the return of learned fear via reinstatement, renewal, or spontaneous recovery. It was believed that extinction training, while the CS–US association was still labile, provided updated information regarding the association between the CS and US before the original association had been consolidated. This finding was not well replicated in rodents [see Ref. (88, 89)] and was only weakly observed in humans [see Ref. (90)].

More recently, Monfils et al. (33) developed a paradigm termed retrieval + extinction in which a single CS+ trial is presented without the US to open a reconsolidation window in which the original fear memory is returned to a labile state. Extinction training is then administered within this reconsolidation window (e.g., 1 h) in an effort to disrupt reconsolidation of the original fear memory. Monfils and colleagues showed that retrieval + extinction effectively attenuated the return of fear through renewal or reinstatement (33). Following this work, Schiller et al. (59), using skin conductance measures, showed that retrieval + extinction (with 10 min but not 6 h between sessions) attenuated spontaneous recovery and reinstatement of fear in human subjects, an effect that was evident a year after the original acquisition of fear.

Similar to the aforementioned studies using immediate versus delayed extinction, attempts to replicate the retrieval + extinction effects reported by the Monfils et al. (33) and Schiller et al. (59) groups have yielded mixed results in human studies. It has become apparent that there are specific boundary conditions under which retrieval + extinction is effective. These conditions include, but are not limited to, the type of psychophysiological measure employed, the schedule of reinforcement used during acquisition, the fear-relevancy of the conditioned stimuli presented, the strength of the fear memory following acquisition, and the presence or absence of on-line measures of expectancy or fear ratings [see Ref. (37, 91–93)].

KEY CONCEPT 8. Boundary conditions Boundary conditions are the conditions under which fear conditioning paradigms (e.g., retrieval ⩲ extinction) are effective. For example, these conditions include, but are not limited to, the type of psychophysiological measure employed (e.g., skin conductance), the schedule of reinforcement used during acquisition, and the strength of the fear memory following acquisition.

## Conclusion

The utility of fear conditioning-related intermediate phenotypes can be observed across multiple units of analysis. Understanding these phenotypes (i.e., fear inhibition, high fear load, fear generalization, etc.) and the divergent manifestations of these responses in the context of a broader etiology (i.e., genetic, behavioral, symptomatic, and clinical studies) may lead to more effective treatment and preventative strategies for PTSD and other trauma-, stressor-, and anxiety-related disorders.

While the present review summarized the current progress of interfacing fear conditioning-related intermediate phenotypes and genetic (54), behavioral (47), symptomatic (15, 45), and clinical studies (78), further characterization of these intermediate phenotypes will enhance the utility of fear-conditioning studies. For example, PTSD is known to affect women more commonly than men (1, 94), and fear-conditioning paradigms are useful for characterizing the sex differences underlying different neuronal, endocrine, and behavioral responses to trauma and other stressors. For example, a polymorphism in the pituitary adenylate cyclase-activating polypeptide receptor gene (*ADCYAP1R1*) is associated with decreased fear discrimination in females but not males (95). These findings illustrate the association between genetic risk, brain–behavior interactions, and intermediate phenotypes.

Ultimately, potential breakthroughs will increase the utility of fear-conditioning studies as a translational platform for studying the intermediate phenotypes underlying dysregulated fear learning, a central tenant of trauma-, stressor-, and anxiety-related disorders. This is especially useful due to the high comorbidity of PTSD and other disorders including depression and substance abuse. By identifying these intermediate phenotypes, we can create specific testable measures, which can then be used to examine the effectiveness of treatment interventions.

## Conflict of Interest Statement

The authors declare that the research was conducted in the absence of any commercial or financial relationships that could be construed as a potential conflict of interest.
